# Identification of a gene signature for different stages of breast cancer development that could be used for early diagnosis and specific therapy

**DOI:** 10.18632/oncotarget.26448

**Published:** 2018-12-21

**Authors:** Charu Kothari, Geneviève Ouellette, Yvan Labrie, Simon Jacob, Caroline Diorio, Francine Durocher

**Affiliations:** ^1^ Département de médecine moléculaire, Faculté de médecine, Université Laval, Québec, Canada; ^2^ Centre de recherche sur le cancer, Centre de recherche du CHU de Québec-Université Laval, Québec, Canada; ^3^ Laboratoire de pathologie, Hôpital du Saint-Sacrement, CHU de Québec-Université Laval, Québec, Canada; ^4^ Département de médecine sociale et préventive, Faculté de médecine, Université Laval, Québec, Canada

**Keywords:** human transcriptome array (HTA) analysis, breast cancer progression, invasive ductal carcinoma (IDC), ductal carcinoma *in situ* (DCIS), gene signature

## Abstract

Breast cancer (BC) is a heterogeneous disease where the survival rate of patients decreases with progression of the disease. BC usually has a linear progression, classified into normal/benign, atypical ductal hyperplasia (ADH), ductal carcinoma *in situ* (DCIS), and invasive ductal carcinoma (IDC). This study aimed to identify gene signature for each of these subgroups.

We performed human transcriptome array analysis on 5 patient samples from each Normal, ADH, IDC and DCIS and 2 replicates of MCF10A cell line representative of each subgroup.

We identified *SFRP1* and snoRNAs (especially *SNORD115* and *SNORD114*) as the initial regulators of cancer progression, accompanied by significant changes in extracellular matrix organization. Tumor progression to the IDC stage showed upregulation of tumor promoting genes responsible for increased invasion, inflammation, survival in stress environment and metastasis.

The gene signatures identified in this study could represent potential biomarkers for each subgroup of breast cancer progression, which could assist in early diagnosis of breast cancer progression as well as treatment interventions. Moreover, these gene signatures could serve in discovery of specific targeted therapies for each subgroup.

## INTRODUCTION

Breast cancer (BC) is the most common cancer in women and the leading cause of cancer-associated death among women worldwide [1]. According to 2017 data of BC Organization, one out of 8 U.S. women (around 12%) will be diagnosed with invasive BC in their life time, thus constituting 25% of all new cases of cancer reported and 13% of cancer-associated death [2]. BC is a complex disease, represented by a collection of tumors with different behavior and clinical outcome, reflecting the biological heterogeneity and difference of genetic changes.

BC can be divided in two major histological subtypes, namely ductal carcinomas and lobular carcinomas, for which a large proportion (about ≥75%) is diagnosed as ductal carcinomas [3]. The initial diagnosis of BC relies on mammography, epidemiological data and morphological observations. Based on these parameters, a simple and linear BC progression is suggested and attributed to a continuum of epithelial cell transformation starting from atypical ductal hyperplasia (ADH) to ductal carcinoma *in situ* (DCIS), both conferring an increased risk of developing invasive ductal carcinoma (IDC) [4]. On the other hand, several studies have proposed two separate pathways of progression which are characterized by 16q loss (in low grade tumors) and second by amplification of 11q13 and 17q1 [5]. Additionally, studies have shown that there is predisposed genetic alterations in the low or intermediate grade tumors that could lead them to any of these pathways [6]. Reports suggest that the degree of increased BC risk depends on the specific epithelial abnormality, pointing toward IDC as possibly originating from benign diseases [7]. The major problem is to identify which benign disease will eventually transform into IDC, so that an early preventive measure could be taken in these patients.

Studies on BC evolution from benign lesions to invasive disease have been facilitated by the use of well characterized cell lines, such as the MCF10A series [8, 9]. These cells provide a tool to study the different stages of BC development between benign lesions, ADH, DCIS, and malignant cells able to form tumors with metastatic capabilities.

Many studies have been carried out in an attempt to identify biomarkers of BC [10, 11]. These studies identified gene expression signatures or other kinds of genetic alterations such as epigenetic signatures, loss of heterozygosity and allelic imbalance resulting from the development of malignancy. Studies have also identified alterations in various gene loci (loss and gains) resulting in dysregulated gene expression observed in BC tissues classified according to their molecular subtypes [12]. Still, early markers to identify benign or premalignant diseases likely evolving into breast cancer are missing.

In the present study, we have identified preventive molecular biomarkers in breast lesions of varying severity and MCF10A cell line series, which could likely be involved in the progression and transformation of premalignant breast lesions into IDC. These early biomarkers of IDC predisposition are deeply needed in prevention and would be a huge step further in clinical settings. Although numerous studies investigated the role of specific genes in different stages or transitions of cancer progression, to our knowledge this study represents the first gene expression analysis performed in a whole continuum of breast lesions.

## RESULTS

### Gene expression signatures clustered according to their stage of BC progression

**Figure 1:**
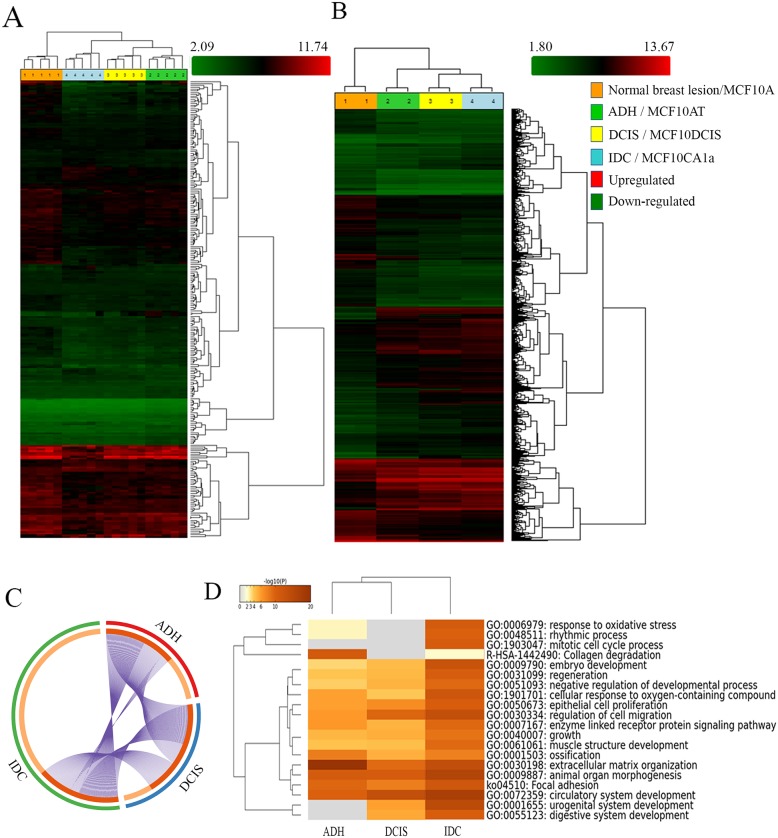
Analysis of BC progression by Human Transcriptome analysis and Metascape (http://metascape.org). Hierarchical clustering of breast lesions **(A)** and representative MCF10A cell lines **(B)** based on 255 and 2800 differentially expressed gene isoforms, respectively (± 1.5-fold and p < 0.05). Clustering analysis was performed using the Transcriptome Analysis Console (TAC) Software (Thermo Fisher Scientific, Canada). The circos plot showing the gene distribution (± 1.5-fold and p < 0.05) of differentially expressed genes in the three subgroups (ADH, DCIS and IDC) as compared to Normal breast lesions **(C)**. On the outside, each arc represents the identity of each gene list (ADH= Red, DCIS= Blue and IDC= Green). On the inside, each arc represents a gene list, where each gene has a spot on the arc. Dark orange = genes in multiple lists; Light orange = unique to the list. Purple lines link the same genes that are shared by multiple gene lists. Enrichment Ontology cluster across the study **(D)** depicting statistically enriched pathways clustered based on Kappa-statistical similarities (Kappa score = 0.3). The colour of the heatmap depicts their p-values, white cells = lack of enrichment. Normal breast lesion: benign breast tissue; ADH: Atypical ductal hyperplasia; DCIS: Ductal carcinoma *in situ*; Invasive: Invasive ductal carcinoma. MCF10A: non-tumorigenic, non-metastatic; MCF10AT (atypical): tumorigenic, non-metastatic; MCF10DCIS (Ductal carcinoma *in situ*): tumorigenic; locally invasive, non-metastatic and MCF10CA1a (invasive): metastatic.

Ductal breast disease represents ≃75% of all diagnosed breast diseases and it is further divided into ADH, DCIS, and IDC, according to its morphological and histopathological features. Besides its prevalence, the molecular signature associated with each stage is not well established. To identify the gene signature which could differentiate each subgroup of BC development, we performed HTA analysis on breast lesions of varying aggressiveness (5 samples/group) namely Normal (Benign), ADH, DCIS and IDC. We also incorporated in our study MCF10A cell lines, which is a well-established *in vitro* model widely used in BC research. As displayed in Figure 1, gene expression data showed hierarchical clustering of each sample according to their subgroup. Samples from a given subgroup clustered distinctively together, demonstrating the reliability of breast tissue selection and molecular characterization. We identified 255 genes differentially expressed in breast lesions (Figure 1A) and 2800 genes differentially expressed in MCF10A cell line series (Figure 1B) (both ANOVA p < 0.05).

### Gene enrichment ontology highlighted extracellular matrix re-organization and response to cellular stress as key factors in BC progression

The distinct clustering of breast tissue samples according to their subgroup led us to further evaluate their gene enrichment ontology. We performed comparison analysis (metascape server) of all differentially expressed genes by comparing the expression profile of each subgroup to that of normal (ANOVA p < 0.05; fold difference ±1.5). The circos plot depicted in Figure 1C shows that the total number of genes whose expression was altered in the IDC subgroup (Green) was very distinct from ADH and DCIS subgroups when compared to normal (Figure 1C), and this was further confirmed by TAC software analysis. The comparison of expression patterns of these genes showed that a significant number of gene isoforms were over-expressed in IDC when compared to normal subtype in both breast lesions (Supplementary Figure 1A) and MCF10A cell lines (Supplementary Figure 1B), as depicted by scatter plots.

In attempt to further classify the changes in gene ontology, which could suggest characteristic features to each subgroup, we identified in Figure 1D statistically significant enriched ontology for each subgroup according to the changes in the hallmark gene set identified by HTA analysis (ANOVA p < 0.05; fold difference ±1.5).

The results described in Supplementary Figure 2A showed the network of the gene ontology cluster identified. The network is visualized with Cytoscape (v3.1.2) with “force-directed” layout and edge bundled for clarity. The same enrichment network was displayed as pies in Supplementary Figure 2B. The enrichment cluster (Supplementary Figure 2A and 2B) highlighted significant gene enrichments related to collagen degradation (R-HAS-1442490), extracellular matrix organization (GO:0030198) and focal adhesion (ko04510) in ADH, whereas genes related to extracellular matrix organization (GO:0030198), epithelial cell proliferation (GO:0050673) and regulation of cell migration (GO:0030334) were enriched in DCIS. Genes related to response to oxidative stress (GO:0006979), mitotic cell cycle process (GO:1903047), extracellular matrix organization (GO:0030198), focal adhesion (ko04510), epithelial cell proliferation (GO:0050673) and regulation of cell migration (GO:0030334) were enriched in IDC. Similar analysis with Panther classification system software showed enrichment of genes associated with catalytic activity, receptor activity and transporter activity in ADH, whereas DCIS has enrichment of genes involved in catalytic activity and enzyme regulatory activity. As for IDC, it showed enrichment of genes related to structural molecular activity, nucleic acid binding transcription factor activity and receptor activity (Supplementary Figure 3). The results obtained from these two analysis tools complement each other, further confirming our analysis.

**Figure 2:**
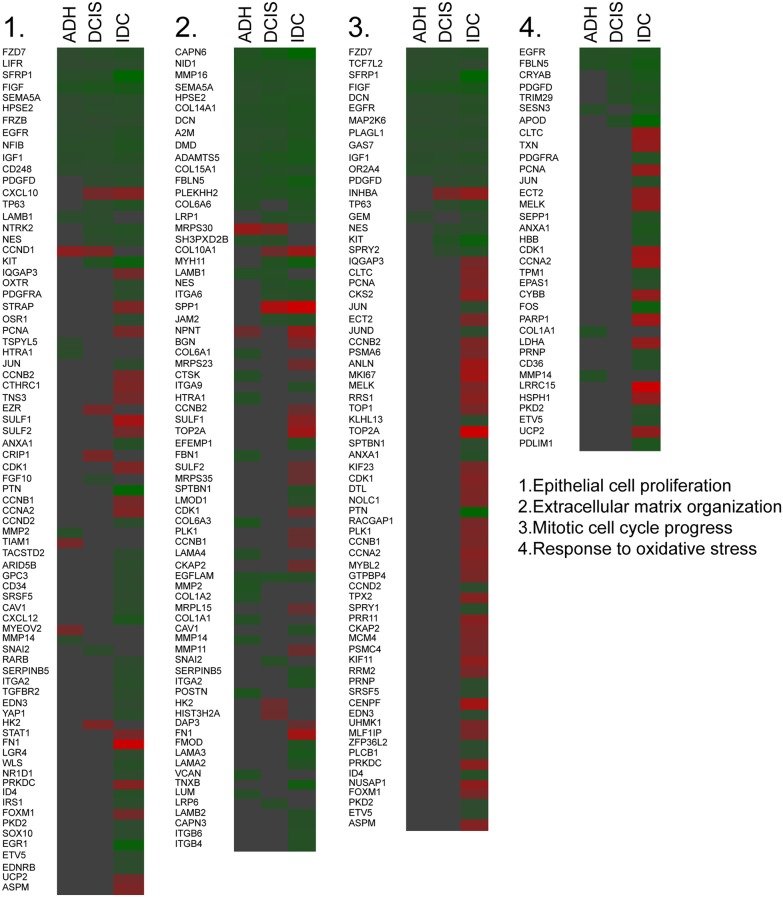
Changes in genes responsible for the gene annotation Heat map indicating the fold change of the gene expression in each subgroup based on the enriched ontology (Green = Downregulation, Red= Upregulation).

Further, we verified the fold difference in the expression level of genes enriched for a particular gene ontology identified by metascape server in breast lesion samples. The fold difference indicated an increase in genes involved in epithelial cell proliferation (23.37%), extracellular matrix organization (24.65%), mitotic cell cycle progression (53.52%) and response to oxidative stress (37.14%) in IDC (Figure 2) as compared to other subgroups. Similar results were also found in MCF10A cell line series (data not shown).

### IPA analysis identified major regulatory pathways for each subtype of BC progression

**Figure 3:**
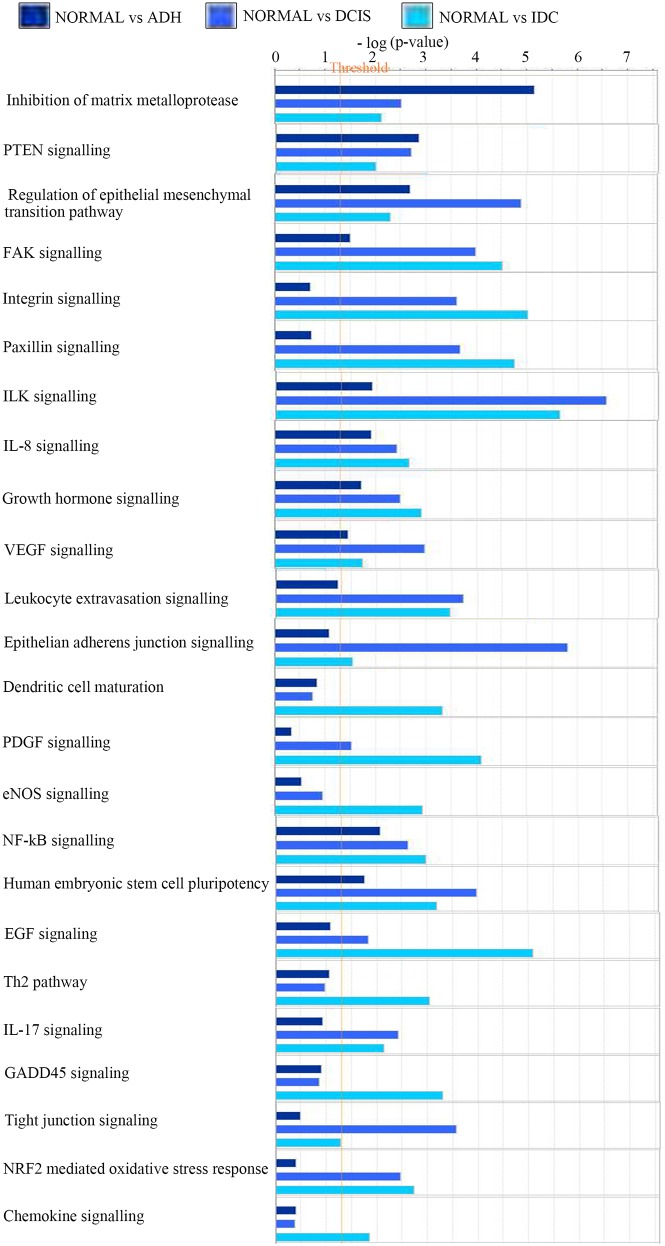
Changes in pathways responsible for the gene annotation Bar chart showing the changes in the canonical pathways in the three subgroups as compared to normal breast lesions. Normal: benign breast tissue; ADH: Atypical ductal hyperplasia; DCIS: Ductal carcinoma *in situ*; IDC: Invasive ductal carcinoma.

Using IPA, the comparison analysis of gene expression fold changes of each subgroup when compared to normal tissue allowed the identification of potential deregulated pathways. As displayed in Figure 3, the analysis revealed a significant upregulation of genes leading to inhibition of matrix metalloproteases in ADH (5.2-fold; p value=7.01E-06) and PTEN signaling (2.9-fold; p-value=1.43E-03), which is not observed in transition to DCIS and IDC. In addition, regulation of epithelial to mesenchymal pathway (4.9; p-value=1.29E-05), epithelial adherens junction signaling (5.8; p-value=1.57E-06), VEGF signaling (3; p-value=1.1E-03) and actin cytoskeleton signaling (4.25; p-value=5.66E-05) were significantly upregulated in the normal-DCIS transition. Moreover, for the normal-IDC transition, a significant increase in EGF signaling (5.1; p-value=8.4E-06), GADD45 signaling (3.3; p-value=4.9E-04), Th2 pathway (3.03; p-value=9.2E-04), PDGF signaling (4.1; p-value=8.2E-05), eNOS signaling (3; p-value=1.2E-03), dendritic cell maturation (3.3; p-value=4.8-04) and chemokine signaling (2; p-value=1.4E-02) was observed. Further, IPA analysis also highlighted a significant increase in the number of genes affecting cellular growth and proliferation, cellular development and cellular movement in a linear fashion from ADH to IDC (Supplementary Table 1).

In addition, a few pathways showed an increasing trend along BC progression from normal to IDC state. FAK signaling (1.5-4.5, ADH to IDC; p-value=3.03E-05), integrin signaling (0.7-5; p-value=9.7E-0.6), paxillin signaling (0.07-4.7; p-value=1.7E-05), ILK signaling (2-5.6; p-value=2.3E-06), growth hormone signaling (1.7-3; p-value=1.2E-03), NFkB signaling (2-3; p-value=1E-03), NRF2 mediated oxidative stress response (0.4-2.73; p-value=1.9E-03) and IL-17 signaling (0.9-2.13; p-value=7.3E-03) all showed an increasing regulation.

### Gene signature for each subgroup

**Figure 4:**
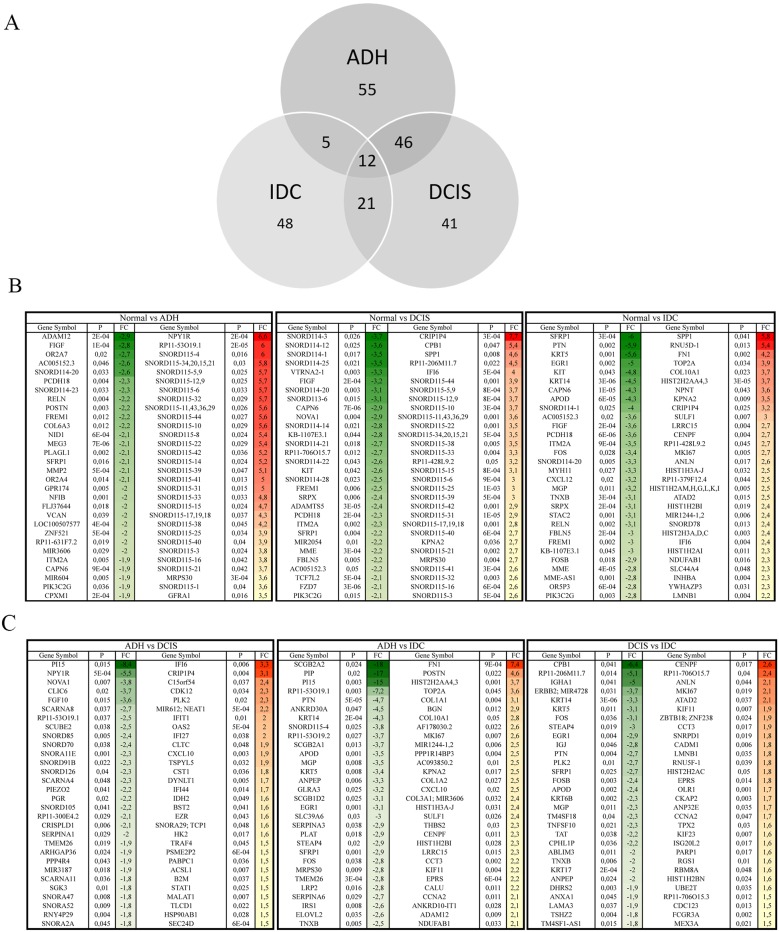
Comparison of the gene expression among different subgroups Venn-diagram **(A)** representing differentially expressed ncRNAs in each subgroup of BC progression as compared to normal breast lesions. **(B)** The top 30 (± 1.5-fold and p < 0.05) differentially regulated genes when compared to gene expression in normal breast lesions or inter groups comparisons **(C)**. Green = Downregulation, Red= upregulation. Normal: benign breast tissue; ADH: Atypical ductal hyperplasia; DCIS: Ductal carcinoma *in situ*; IDC: Invasive ductal carcinoma.

In our study we found genes whose expression was changed as the normal cell progresses towards different stages of cancer development. In addition to coding genes, various non-coding RNAs (ncRNA), whose expression was significantly altered, were also identified (Figure 4A). A total of 55, 41 and 48 differentially expressed ncRNAs in ADH, DCIS and IDC subgroup, respectively, were identified (Figure 4A). Particularly, ncRNAs such as *SNORD115*, *SNORD116*, *SNORD114*, *SNORD113*, *SNORD78* and *miR205* were highlighted in this study. Corresponding gene signatures were then established for each transition. *SNORD115* (*SNORD116* for MCF10A cell line) gene cluster was found as a potential specific gene signature through ADH and DCIS subgroups (Figure 4B). In order to understand the role of SNORD115 and SNORD116 in tumor development, we analyzed data obtained from Falaleeva et al. [13], which showed SNORD115/116 as the initial regulator of gene expression, directing the cell towards an invasive phenotype (Supplementary Figure 4). A decrease in expression level of *SNORD114*, *SFPR1* and *PI15* was observed with tumor development (Figure 4B, 4C). Furthermore, *SPP1, FN1, TOP2A, ANLN, POSTN, CENPF, LRRC15,* and *SNORD78* gene expression and other genes related to invasion, extracellular matrix organization and epithelial cell cycle progression, mitotic cell cycle progression and response to oxidative stress showed a significant upregulation in IDC sub-group (Figure 2, 4B, 4C). It should be noted that a similar expression pattern was also found in MCF10A cell lines for some of these genes (data not shown).

**Figure 5:**
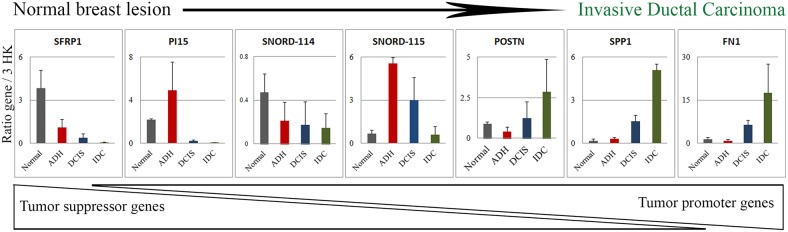
Validation of the difference in gene expression by qPCR Based on the changes in the top 30 genes depicted in Figure 5B and 5C, 7 genes were selected and validated by qPCR in tissue samples (N=3). The expression data is the ratio of query gene to 3 housekeeping genes (*ATP50, HPRT1* and *GAPDH*). The graph is representative of two independent experiments. Normal: benign breast tissue; ADH: Atypical ductal hyperplasia; DCIS: Ductal carcinoma *in situ*; IDC: Invasive ductal carcinoma.

Seven genes namely *SFRP1, PI15, SNORD114, SNORD115, POSTN, SPP1* and *FN1* identified as significantly deregulated among subgroups were then selected from the top 30 genes (Figure 4B, 4C) for quantitative PCR (qPCR) validation (Figure 5). As depicted in Figure 5, the qPCR validated the accuracy of our findings. A similar expression pattern of these genes in MCF10A cell lines was indicated in Supplementary Table 2.

## DISCUSSION

The identification and characterization of a breast tumor is routinely performed based on morphological and histopathological features. The quantitative analysis of DCIS lesions differentiating them from ADH lesions is based on the presence of a homogenous population in at least two membrane bound spaces with a size of more than 2mm [14], making the identification difficult. It has been shown that 30% of ADH upgrade to DCIS or IDC by the time of surgery [15] and 30% of DCIS upgrade to IDC [16]. Efforts have been undertaken to develop a clinical test [17] that could predict which patient has a predisposition regarding factors that could lead to transformation of benign or low grade tumours to IDC, but so far none has been successful in clinical setting. So far, molecular gene signatures that could identify the early changes in BC progression remain to be elucidated. To identify a subset of gene signatures which could be associated with a specific pathological subgroup of BC progression continuum, we carried out HTA analysis on each subgroup namely Normal, ADH, DCIS and IDC. We observed a total of 255 genes differentially expressed when considering all different types of breast lesions, and 2800 genes in MCF10A cell lines were identified. Results suggested tremendous changes in gene expression levels of several genes/pathways, which correlated with the transformation of breast cells from normal to IDC. Indeed, a total of 231 genes (out of 255 differentially expressed genes) were deregulated in IDC when compared to normal cells in breast tissue samples while 1595 genes (out of 2800 differentially expressed genes) were significantly dysregulated in MCF10A cell line series between normal and invasive.

Gene expression data highlighted that during the transformation of breast cells from Normal to ADH and then to DCIS stage, the major biological functions involved were extracellular matrix organization and signaling, which includes the enzymatic activity to degrade the extracellular matrix as a preparatory step to matrix invasion. This data is in concordance with a previous studies. [3, 18]. An increase in the angiogenesis-related pathways [19] such as changes in the extracellular matrix, endothelial cell proliferation, migration, VEGF signaling, and actin cytoskeleton signaling at the DCIS stage was also observed. Once the cells were prepared to switch to the IDC stage, in addition to the genes involved in extracellular matrix re-organization and epithelial cell proliferation, upregulation of genes involved in stress environment, i.e. genes involved in survival in a stress environment (eNOS pathway, NRF2 mediated oxidative response, GADD45 pathway etc), inflammatory processes (Th2 pathway, chemokine signaling, NFkB pathway, IL-17 signaling), growth factor signaling (EGF signaling) and transcription initiation and mitosis (*TOP2A*) was observed. These data are in concordance with the literature, which highlights an increase in inflammatory processes [20], DNA replication [21] and survival in stress environment [22] to be associated with BC progression. Furthermore, we have also observed a linear progression in the cellular functions (cell growth, proliferation, development and movement) as the tumor develops from ADH to IDC, and this is also shown by a study by Hou et al., 2016 [23].

In addition, a significant number of non-coding RNAs (ncRNAs) were pinpointed as significantly modulated (Figure 5A). Many studies highlighted the role of ncRNA in BC initiation and progression [24, 25]. In our study, the more significant regulated ncRNAs were *SNORD115* (breast lesion)*, SNORD116* (MCF10A)*, SNORD114, SNORD78* and *miR205.* SNORD proteins are members of the non-coding small nucleolar RNA (sno-RNA) family with C/D box and are associated with DNA methylation. This gene family usually found in gene clusters, has roles in RNA nucleotide modification and mRNA splicing [26]. Sno-RNA can also generate miRNAs which can affect expression of many genes [27]. Deregulation of sno-RNA expression has been seen in many cancers [24, 25]. Kishore and Stamm reported that *SNORD115* was essential for correct splicing of a serotonin receptor *Htr2c*, whose expression has been correlated with breast tumor progression [26]. Our results showed an increase in *SNORD115* in ADH and DCIS, and a similar modulation of *SNORD116* was seen in MCF10A cell lines. Reports suggest that *SNORD115* and *SNORD116* affect each other's activity and hence modulate the expression of their target genes [13]. As previously published, Falaleeva et al (2015) showed that the over-expression of *SNORD115* and *SNORD116* altered the pathways related to cellular response to DNA damage, regulation of cellular response to stress, post transcriptional regulation of gene expression, mitotic cell cycle progression, cytokine signaling and TNF signaling [13]. This is in agreement with our results characterizing the Normal to IDC transition in breast tissue lesions. These data display the initial role of SNORD115/116 as a preparatory signal for cancer progression towards IDC. A decrease in *SNORD114* expression, which is located at the *MEG3-MEG8* loci in the genome, was also observed in our study [28, 29]. Previous studies have shown a suppression of *MEG3* gene in various tumors [30, 31] and a downregulation of *MEG8* gene, which is essential for stem cell growth and proliferation, thus resulting into a cancer cell [29]. In addition, we have found an increase in *SNORD78* (2.4 fold, p-value = 1.3E-03) and a decrease in miR205 expression (-2.5 fold, p-value = 1.6E-03) in IDC subgroup when compared to normal, which are in concordance with the literature, suggesting that *SNORD78* expression was associated with tumorigenesis [32], while miR205 could supress cell growth and invasion in BC [33]. Validation of *SNORD115* and *SNORD114* gene expression by qPCR in breast tissue lesions confirmed these modulations. MCF10A cell lines also showed similar patterns.

**Figure 6:**
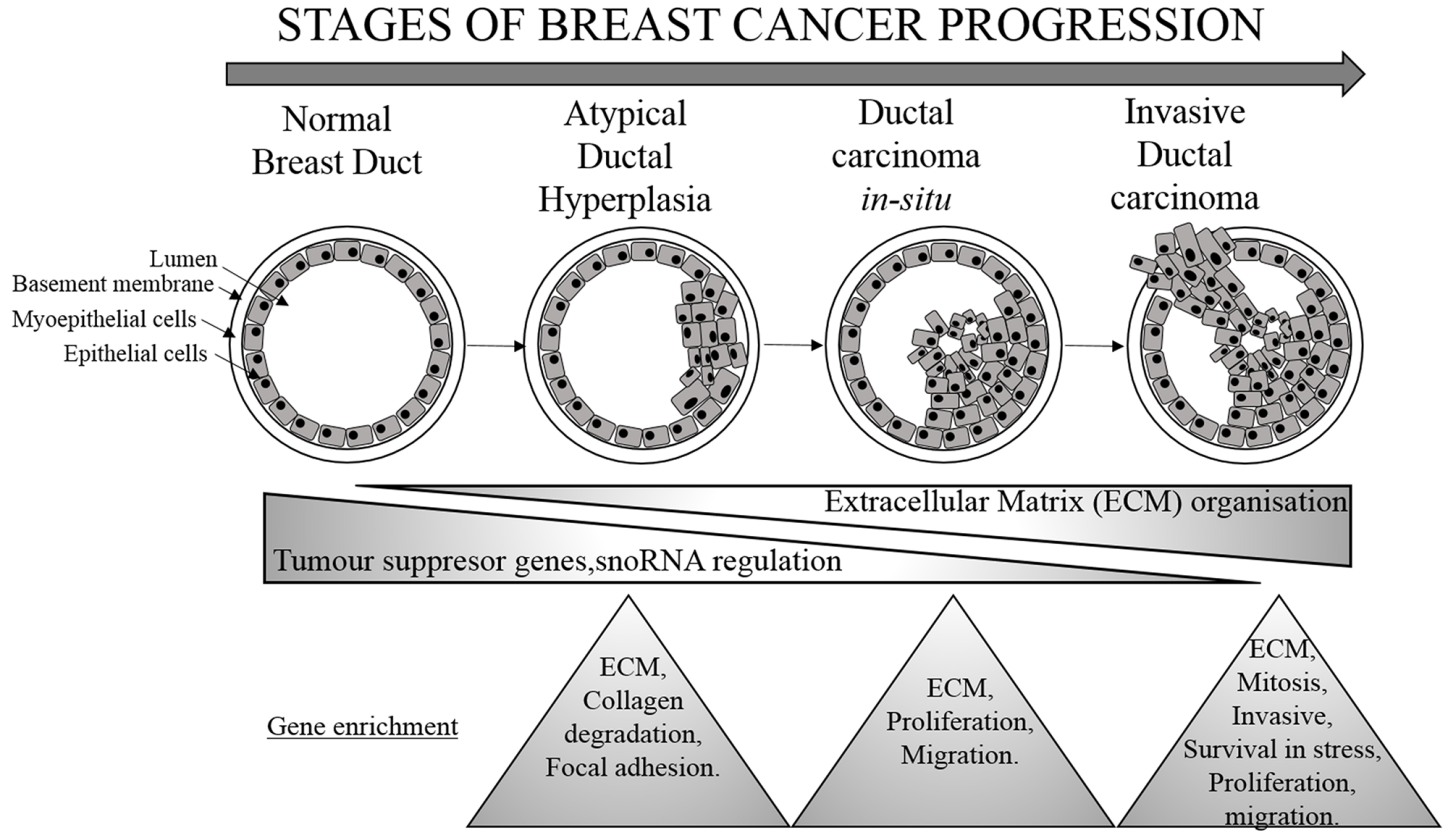
Molecular changes associated with breast cancer progression Stages of BC progression where decrease in tumor suppressor gene, snoRNA regulation and increase in genes responsible for extracellular matrix organisation is associated with breast cancer progression. The triangles represent the changes at each subgroup of BC progression.

In addition to ncRNAs, we found differentially regulated protein coding genes belonging to molecular pathways responsible for BC progression. Indeed, *SFRP1, PI15, RELN, POSTN, SPP1, FN1, TOP2A, ANLN, CENPF* and *LRRC15* could represent potential signatures corresponding to each subgroup. Out of these, *SFRP1, PI15* and *RELN* were downregulated as the tumor progresses toward malignancy. On the other hand, *POSTN, SPP, TOP1, ANLN, CENPF* and *LRRC15* were upregulated at the IDC stage (Figure 6).

Four of these genes namely *SFRP1, PI15, SPP1* and *FN1* have been then confirmed by qPCR. We observed a significant decrease in *SFRP1* as the tumor cancer progresses towards IDC. The SFRP1 (Secreted Frizzled Related Protein 1) protein is a negative regulator of the Wnt pathway [34]. As described earlier, this protein harbours a cysteine rich domain which is homologous to frizzled receptor [34]. After being secreted by the cell the protein remains associated with the membrane and can interact with other proteins in the extracellular space. Upon binding to the WNT protein, SFRP1 inhibits the ligand receptor binding and hence circumvents WNT signaling which therefore behaves both as oncogenic as well as a tumor suppressor signal depending on the context. In BC, downregulation of SFRP1 protein is associated with tumor progression and poor prognosis [35], which is in agreement with our results. Furthermore, we found that a decrease in *PI15* (Peptidase inhibitor 15) gene correlated with tumor progression. This gene encodes a trypsin inhibitor protein. Interestingly, several studies have shown that food supplements containing trypsin inhibitors might be beneficial in fighting breast and other cancers by inhibiting trypsin used by cancer cells to invade the basement membrane [36].

POSTN (Periostin) is a secreted extracellular matrix glycoprotein which binds to integrins and supports the adhesion and migration of epithelial cells [37]. POSTN has a role in tissue development, regeneration, wound healing as well as epithelial mesenchymal transition [38]. Hence, its increase observed in the invasive subgroup is in agreement with these former observations. POSTN has been reported to play a role in many cancers and has been seen in cancer associated fibroblasts of IDC and high-grade tumors [39] which again is in accordance with our data.

SPP1 (Secreted Phosphoprotein 1) also known as Osteopontin is involved in the attachment of osteoclasts to mineralized bone. It is present in cell membrane and is also a cytokine responsible for upregulation of INFγ and IL-12 [40]. SPP1 is associated with aggressiveness of cancer, increases in tumor promoting inflammation and activates invasion and metastasis [41], which is in perfect agreement with our findings. Based on our data, we found an increase in the inflammatory pathway at the IDC stage. In addition, a significant upregulation of FN1 (Fibronectin1), a glycoprotein found in dimeric or multimeric form in the extracellular matrix, was noted in IDC transition. FN1 is involved in cell adhesion and migration, wound healing, invasiveness and metastasis. In BC, FN1 is secreted by both the stromal and epithelial cells and is a marker of increased invasiveness and metastatic potential [42].

Moreover, other genes were also pinpointed in our study (*RELN, TOP2A, ANLN, CENPF* and *LRRC15)* and were also documented in the literature regarding their expression in BC [43–47].

Downregulation of RELN (Reelin), a secreted extracellular matrix protein, is considered a poor prognosis for BC [43]. *TOP2A* (Topoisomerase (DNA) II Alpha) is involved in chromosome condensation, chromatid separation, and the relief of torsional stress that occurs during DNA transcription and replication. Its upregulation and aberrant expression was observed in many cancers including BC [44]. The upregulation of *TOP2A* in our study can be associated with increased mitotic division seen in the IDC subgroup. ANLN (Anillin Actin Binding Protein), which plays a role in cell growth, migration and cytokinesis, has been shown to be associated with poor prognosis in BC patients and is considered an important player for cell division [45]. As for CENPF (Centromere Protein F), this protein has a major role in chromosome segregation during mitosis and has been associated with chromosomal instability in primary BC patients and therefore poor prognosis [45]. The upregulation of *LRRC15* (Leucine Rich Repeat Containing 15) gene, as observed in IDC when compared to DCIS lesion, is considered to play a major function in BC invasiveness [46]. These studies were in concordance with our findings.

The present study could truly serve in early diagnosis in clinical setting and further characterization could also identify potential and specific targets for each subgroup of BC. Indeed, our analysis highlights involvement of tumor suppressor genes and snoRNAs as initial modulators of breast cell transformation into ADH lesion, signifying bi-directional signaling in the breast cell. As the cell proceeds towards DCIS, extracellular matrix proteins prevail while an increase in signaling associated with increased invasiveness, metastasis, cell division and survival is observed at the IDC stage.

The genes identified in the present study could aid clinicians to make a priority-based decision for intervention. For example, if a patient shows expression of PI15 but lacks the expression of SPP1 and FN1, then this patient could be called for follow-up without the need of immediate surgery. On the other hand, reverse of this situation calls for immediate surgical intervention. The present study provides an opportunity for early detection which is quick and cost-effective in addition further investigation could provide a new and specific target for each subgroup of BC progression.

A study by Hou et al. 2016 [23] has carried out a systemic analysis of genes involved in breast cancer development by carrying out comparative analysis of gene expression in normal, DCIS and IDC stages. This study is however missing one of the important stages of breast cancer progression, that is ADH [48, 49], which is often confused with low grade DCIS. In summary, the present study is the first thoroughly analyzed gene expression in a whole continuum of breast lesions of varying severity, and has led to the identification of a specific human gene signature that could be a potential signature for different stages of cell malignancy development, from normal breast tissue to invasive cells. These genes could therefore be further characterized in breast samples and could be developed as biomarkers for the prognosis of BC progression and invasiveness.

## MATERIALS AND METHODS

### Breast tissue samples

The breast lesion tissue samples were collected from the tissue bank located at the Centre des maladies du sein Deschênes-Fabia of Hôpital du St-Sacrement, Quebec, Canada. All breast tissue samples deposited in the bank were from women (n=20) with no hormonotherapy or chemotherapy treatment before surgery. All breast diseases were confirmed by an experienced pathologist, and all tumor characteristics were routinely collected from medical reports: size, histologic type, grade, lymph node involvement and receptor status (ER, PR and HER2). Formalin-fixed paraffin-embedded (FFPE) blocks containing normal (classified as normal breast tissue), ADH, DCIS or IDC epithelial tissue were selected by a senior pathologist specialized in breast pathologies. Normal tissues were collected from women coming for biopsy. These patients did not had any sign of breast lesions at the time of surgery. High grade DCIS was selected to avoid any contamination with ADH given that ADH and low-grade DCIS share many architectural and cytological features, and risk of invasive BC is higher in high grade DCIS. Since high grade DCIS was selected, we did the same for IDC. Five independent samples corresponding to each stage of BC progression were selected for gene expression analysis. All selected DCIS and IDC were positive for estrogen and progesterone receptors and were of high grade. The mean age of selected women for this study was 53±4 years.

### MCF10A cell lines

The MCF10A cell line subtypes were developed to represent different stages of BC progression [8, 9]. These cell lines were used in parallel to compare *in vitro* MCF10A cell line data with breast lesion clinical data. Two biological replicates of each MCF10A (normal/benign), MCF10AT (ADH), MCF10DCIS (DCIS) and MCF10CA1a (IDC) cells were used for analyses.

### Tissue microarray

For each of the 20 participants, a tissue microarray (TMA) was done to ensure that RNA is assessed on samples containing breast tissue with at least 90% epithelial content. Hematoxylin and eosin (H&E)-staining was performed on first and last tissue sections for review and tissue was validated by an experienced pathologist to ensure consistency of the breast tissue of interest throughout the block (5 normal, 5 ADH, 5 DCIS, 5 IDC).

### RNA isolation

Total RNA from breast tissue samples as well as MCF10A cell lines were isolated with Qiagen RNeasy Mini Kit (Qiagen, Hilden, Germany). Preparation of RNA samples for whole-genome expression analysis was performed using the SensationPlus™ FFPE Amplification Kit (Affymetrix, Thermo Fisher Scientific Waltham, Massachusetts, United States).

### Human transcriptome array analysis (HTA)

HTA analysis was performed on these samples using the GeneChip™ Human Transcriptome Array 2.0 (ThermoFisher Scientific, Massachusetts, United States). HTA hybridization, washing, staining and scanning were performed following the GeneChip™ Human Transcriptome Array 2.0 protocol of the genomic platform located at the CHU de Quebec Research Centre, Laval University.

### Statistical analyses

Identification of differentially expressed gene isoforms between subgroups of lesion or MCF10A cell line series (normal, ADH, DCIS and IDC) was carried out by ANOVA analysis between 4 groups and obtained data were further analyzed with the Transcriptome Analysis Console (TAC) Software (Affymetrix). To do so, normalized intensities between subgroups of lesions or cell lines were compared using One-way Between-Subject ANOVA, and multi-testing correction was performed using Benjamini-Hochberg Step-Up False Discovery Rate (FDR) controlling procedure [50]. Then for statistically significant results, expression analysis was performed for all pairing groups while linear regression was applied for a tissue subtype along the continuum of lesions (normal/ADH/DCIS/IDC) to reveal progressive and significant differences, which is dependent on the lesion aggressiveness level. Finally, the latter expression analysis was repeated regardless of p-values obtained by ANOVA analysis to reveal gene signature for each subgroup. P-values < 0.05 were considered as significant with a FDR of 5%.

### Pathways, networks, and clustering analysis

In order to identify biological pathways differentially expressed between prognostic subgroups, we performed an enrichment analysis of upregulated and downregulated genes (FDR < 0.05) using ClueGO, Metascape server (Sanford Burnham, UCSD, GNF, http://metascape.org) and three different pathways analysis tools: the KEGG (Kyoto Encyclopedia of Genes and Genomes) database, the panther classification system (http://pantherdb.org/) and the Ingenuity Pathway Analysis (IPA®, QIAGEN Redwood City, www.qiagen.com/ingenuity). Default settings in IPA for expression dataset analyses were used for functional analysis. Gene lists were uploaded using NCBI Entrez gene IDs or gene symbols and submitted for IPA Core Analysis. IPA calculates *p*-values that reflect the statistical significance of association between the genes and the networks by Fisher's exact test. *P*-values <0.05 were considered significant. Gene ontology and KEGG pathways enrichment of transcripts significantly and differentially expressed were performed using Gene Set Enrichment Analysis (GSEA) tools with default parameters, while FDR q-values below 0.01 were used.

### Quantitative real-time PCR (q-PCR) analysis

Quantitative PCR was performed using SyBr Green technology as described previously [51]. Briefly, oligo-primer pairs that allow the amplification of ~200 base pairs (bp) of the indicated specific mRNA were designed by GeneTools software and their specificity was verified by blasting the GenBank database. The sequence of primers is indicated in Supplementary Table 3. Data calculation and normalization were performed using the second-derivative and double-correction method [52], with three housekeeping genes (*ATP50*, *HPRT1* and *GAPDH*). The mRNA levels were expressed as number of copies/μg of total RNA calculated using corresponding standard curves.

## SUPPLEMENTARY MATERIALS FIGURES AND TABLES




