# Supplementary material for: Identification of a gene signature for different stages of breast cancer development that could be used for early diagnosis and specific therapy
Source: Oncotarget. 2018 Dec 21;9(100):37407–20. doi: 10.18632/oncotarget.26448 (PMC6324778; doi:10.18632/oncotarget.26448)
Supplement: Supplementary file 1 [file oncotarget-09-37407-s001.pdf]

Identification of a gene signature for different stages of breast cancer development that could be used for early diagnosis and specific therapy

SUPPLEMENTARY MATERIALS

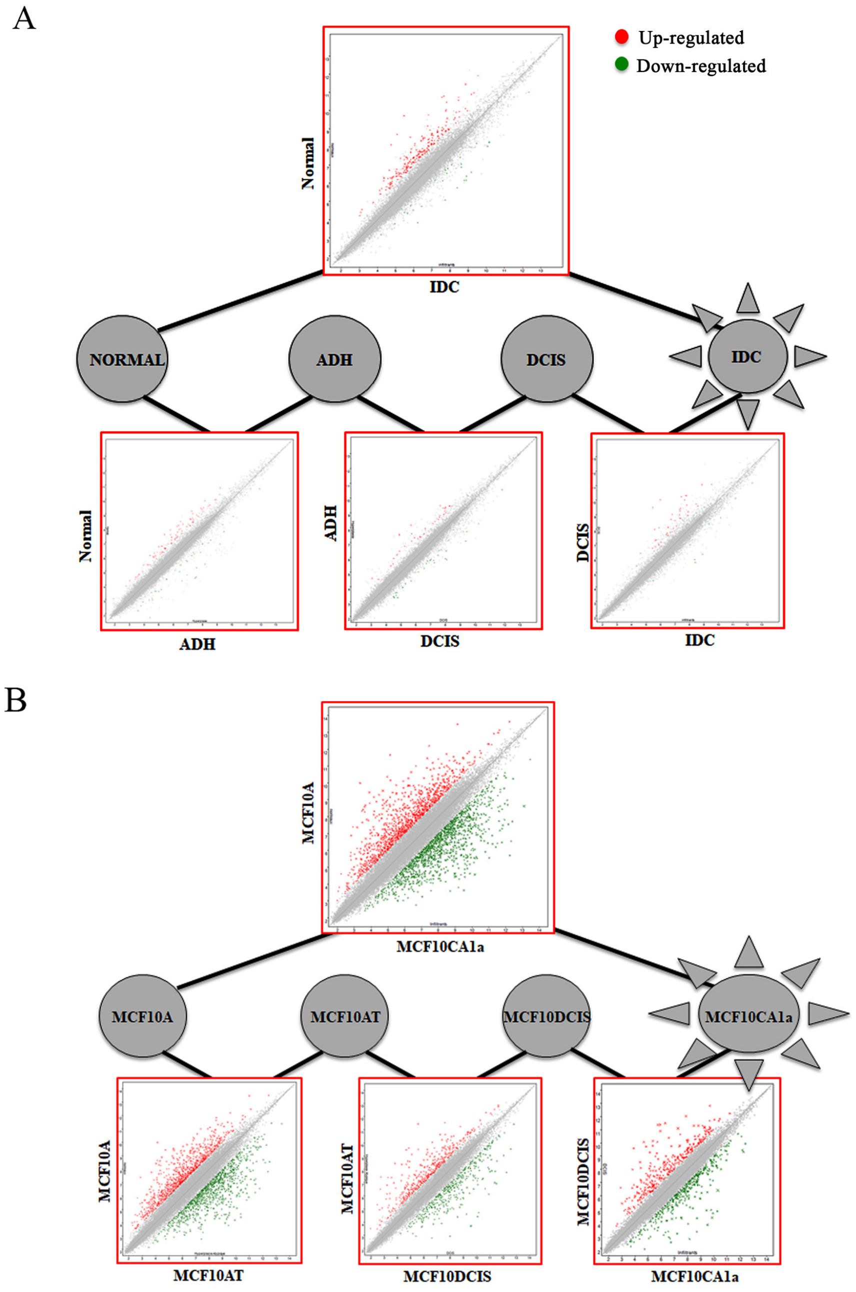

Supplementary Figure 1: Analysis of differentially expressed genes among different sub-groups by TAC software. The number of genes differentially regulated (± 1.5-fold and $p < 0.05$; Green = Downregulation, Red = Upregulation) between different subgroups of breast lesions **(A)** and MCF10A cell lines **(B)** are represented by scatter plots.

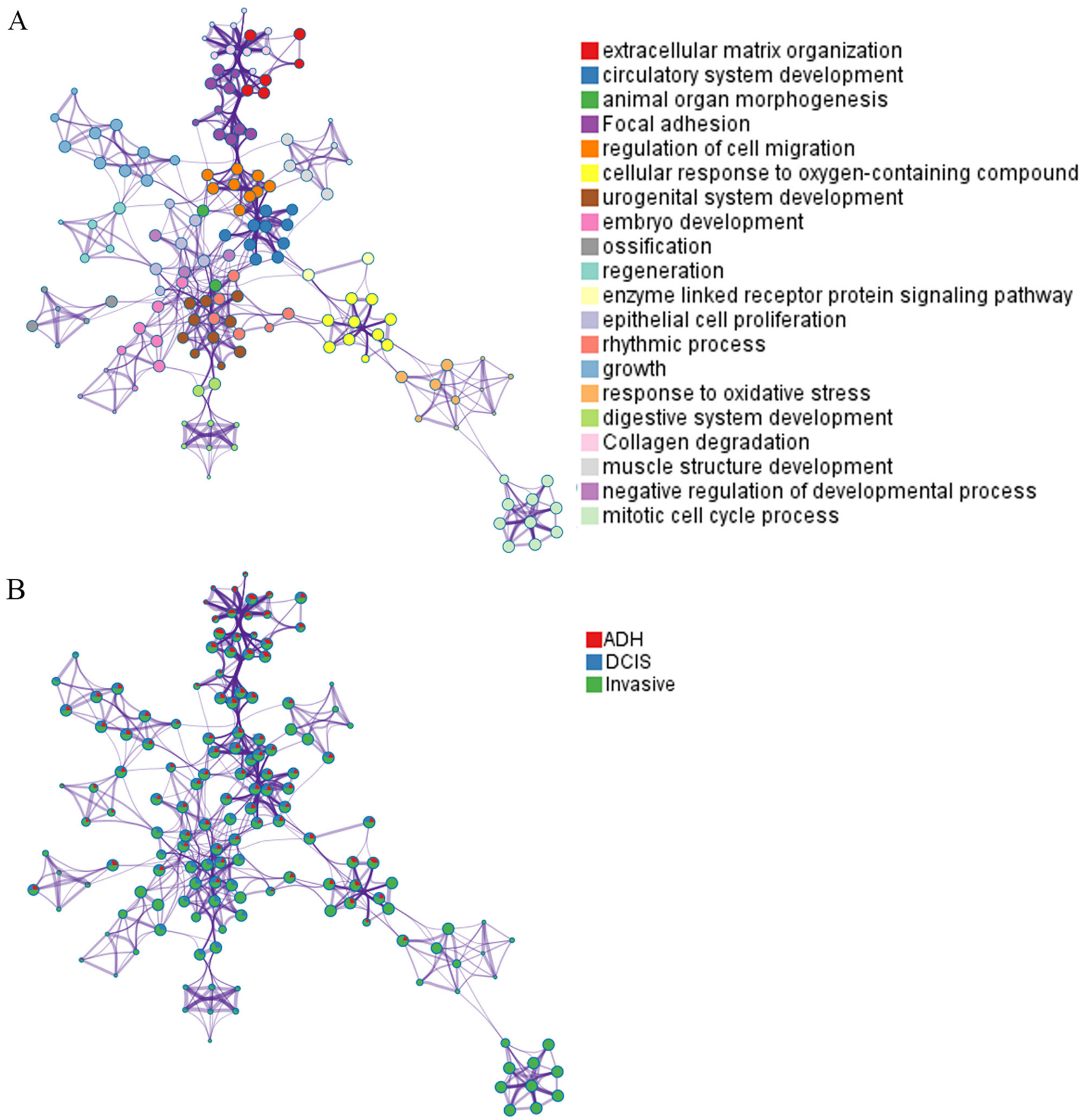

Supplementary Figure 2: Enrichment Ontology Cluster. Based on cluster ID depicting the cluster nodes **(A)** where each gene ontology is represented by a circle node, with its size being proportional to the number of input genes corresponding to a given ontology, while the color represented the cluster identity (i.e., nodes of the same color belong to the same cluster). Terms with similarity score > 0.3 were linked by an edge, where the thickness of the edge represents the similarity score. Depiction of each node by colour coded pie sector **(B)** where each sector represents the number of hits originated from gene list.

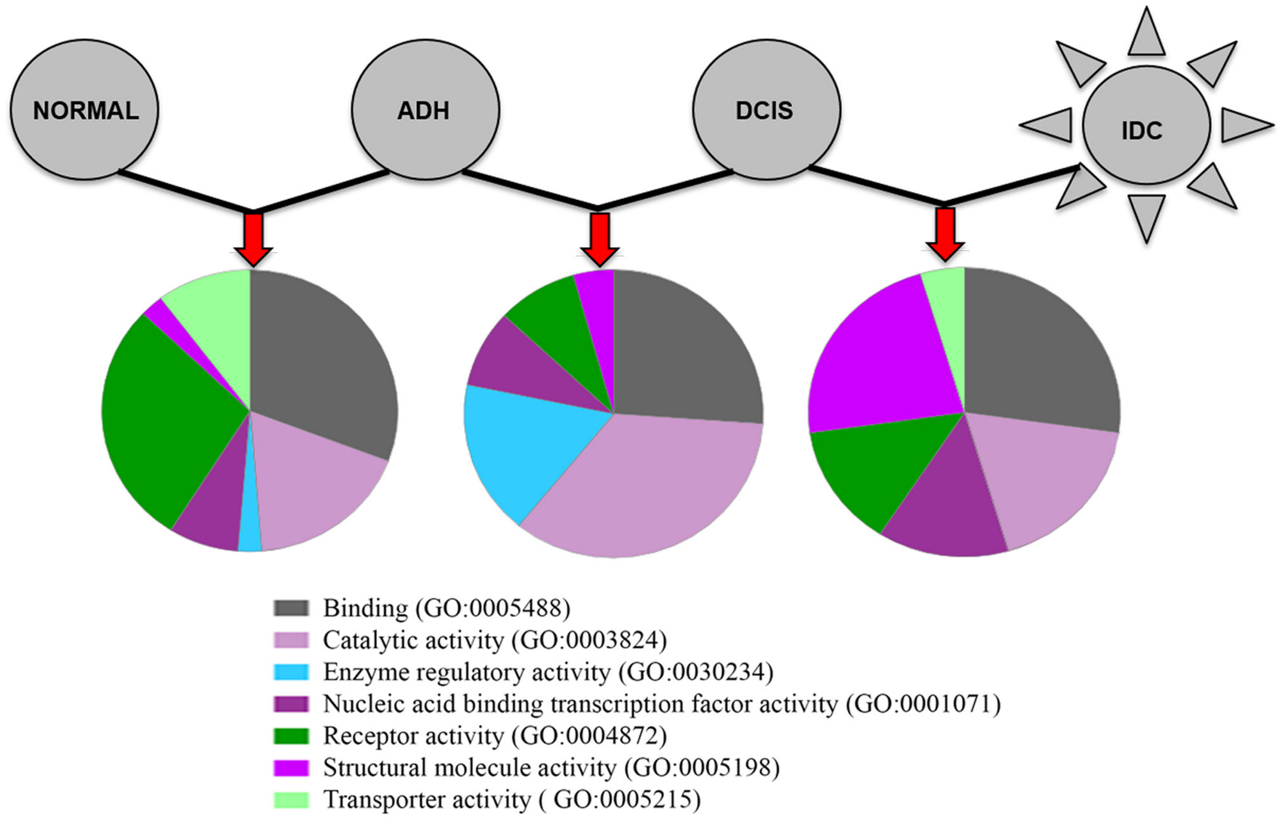

Supplementary Figure 3: Gene Functions of isoforms differentially expressed between breast lesions. Based on GO Molecular function, gene function analysis was performed using Panther classification system (http://pantherdb.org/). A gene function has been annotated to the gene isoforms identified as differentially expressed (± 1.5 fold and $p < 0.05$) between different subgroups of breast lesions. Normal: benign breast tissue; ADH: Atypical ductal hyperplasia; DCIS: Ductal carcinoma *in situ*; IDC: Invasive ductal carcinoma.

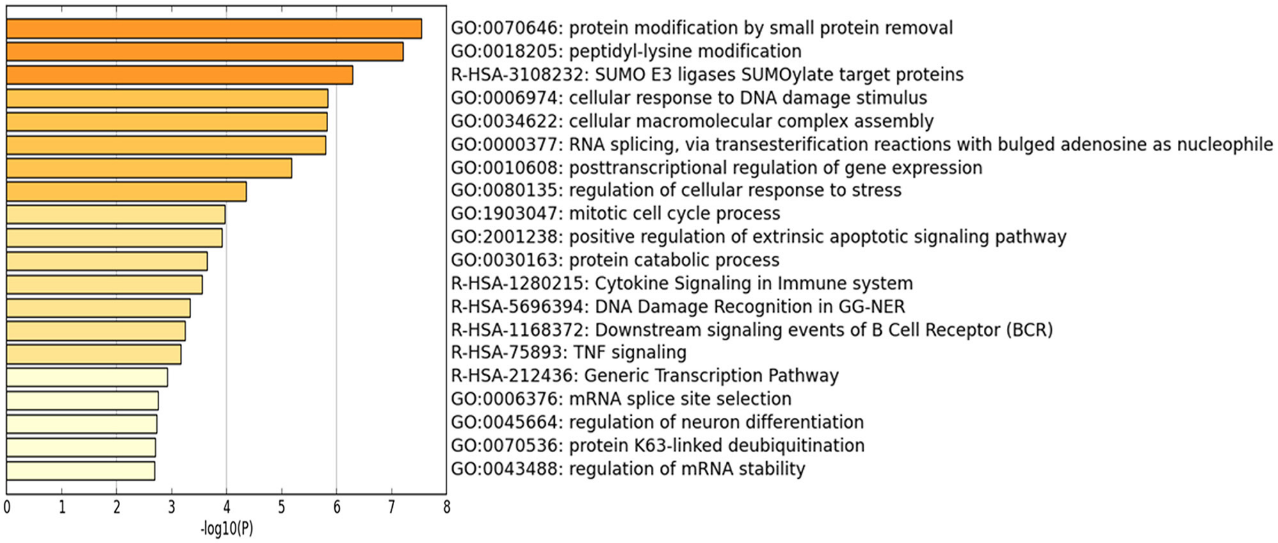

Supplementary Figure 4: Pathways affected by upregulation of *SNORD115* and *SNORD116* (data obtained from Falaleeva et al., [16]). Analysis of Supplementary data 4 obtained from Falaleeva et al. by Metscape server for the genes differentially regulated with over-expression of *SNORD115* and *SNORD116* genes.

Supplementary Table 1: Changes in the cellular functions in the breast cancer continuum

	ADH		DCIS		IDC	
**Cellular functions**	**No. of molecules**	**p-value range**	**No. of molecules**	**p-value range**	**No. of molecules**	**p-value range**
Cellular growth and proliferation	78	4.02E-04 to 2.61E-10	92	1.60E-04 to 3.82E-15	244	9.23E-07 to 7.81E-40
Cellular development	74	4.02E-04 to 2.76E-12	82	1.82E-04 to 3.80E-11	227	9.23E-07 to 5.51E-25
Cellular movement	57	4.41E-04 to 5.61E-12	68	1.62E-04 to 8.80E-15	172	8.97E-07 to 1.33E-32

Supplementary Table 2: Gene expression in MCF10A cell line series as compared to normal MCF10A cell line

Gene Name	MCF10AT	MCF10DCIS	MCF10CA1a
SFRP1	0	-3.36	-4.36
PI15	1.46	-7.43	-4.36
SNORD114	1.15	-1.23	0
SNORD116	4.78	65.97	1.51
POSTN	57.85	98.2	100.83
FN1	5.61	4.53	20.43

Supplementary Table 3: Supplementary Table 3: Primer sequence.

See Supplementary File 1
