# Supplementary material for: Identification of a gene signature for different stages of breast cancer development that could be used for early diagnosis and specific therapy
Source: Oncotarget. 2018 Dec 21;9(100):37407–20. doi: 10.18632/oncotarget.26448 (PMC6324778; doi:10.18632/oncotarget.26448)
Supplement: Supplementary file 2 [file oncotarget-09-37407-s002.docx]

| **Gene Symbol** | **Description** | **GenBank** | **size (bp)** | **Primer sequence 5'→3'**  **S/AS** |
| --- | --- | --- | --- | --- |
| **SNORD115** | Homo sapiens small nucleolar RNA, C/D box 115-11 (SNORD115-11), small nucleolar RNA, all transcripts | NR_003303 | 81 | GGTCAATGATGAGAACCTTATATTGTC/  GGGCCTCAGCGTAATCCTATTG |
| **SNORD114-20** | Homo sapiens small nucleolar RNA, C/D box 114-40 (SNORD114-40), small nucleolar RNA | NR_003213 | 63 | TGGATCGATGATGACTACTGGTG/  AGAGTTCCAGAGACGTATTCACTGTAG |
| **PI15** | Homo sapiens peptidase inhibitor 15 (PI15), 2 transcripts | NM_015886 | 92 | CTTGGGCGGCTACTTGCATTT/  GCGATATCTTCCAGTGCGTACA |
| **POSTN** | Homo sapiens periostin (POSTN), 4 transcripts | NM_006475 | 98 | ATTCCTGATTCTGCCAAACAAGTTATT/  CTCAGAGCAGATGCCAAGCCTAATT |
| **CPB1** | Homo sapiens carboxypeptidase B1 (CPB1) | NM_001871 | 92 | TGCTGGTTGGTGTGAAATTGGAG/  GGGCCTTGGTCTCCTTTTCAG |
| **SPP1** | Homo sapiens secreted phosphoprotein 1 (SPP1), 5 transcripts | NM_001251830 | 108 | AAGAAGTTTCGCAGACCTGACATC/  AACGGGGATGGCCTTGTATGC |
| **SFRP1** | Homo sapiens secreted frizzled related protein 1 (SFRP1) | NM_003012 | 111 | TCTACCCGTGTCGCTGGCTCT/  CCTCGGGGAACTTGTCACACTTAA |
| **FN1** | Homo sapiens fibronectin 1 (FN1), 7 transcripts | NM_212482 | 104 | GCTCAAGTGGTCCTGTCGAAGTAT/  GTACTTGGAAATGTGAGATGGCTGT |
| **ATP5O** | Homo sapiens ATP synthase, H+ transporting, mitochondrial F1 complex, O subunit | NM_001697 | 103 | ATTGAAGGTCGCTATGCCACAG/  CCTTCAGGATTTGTGCTACTCTCA |
| **HPRT1** | Homo sapiens hypoxanthine phosphoribosyltransferase 1 | NM_000194 | 86 | CGTCGTGATTAGTGATGATGAACCA/  ACACCCTTTCCAAATCCTCAGC |
| **GAPDH** | Homo sapiens glyceraldehyde-3-phosphate dehydrogenase | NM_002046 | 93 | CGGGGCTCTCCAGAACATCAT/  ATGCCAGTGAGCTTCCCGTTC |

Supplementary Table 3: Primer sequence
